# COVID-19 Pandemic, Transparency, and “Polidemic” in the Republic of Korea

**DOI:** 10.1007/s41649-021-00164-4

**Published:** 2021-03-12

**Authors:** Cheol Kang, Ilhak Lee

**Affiliations:** 1 Division of Art & Teacher’s Education, University of Seoul, Seoul, Republic of Korea; 2 The Asian Institute of Bioethics and Health Law, College of Medicine, Yonsei University, Seoul, Republic of Korea

**Keywords:** COVID-19, Public health ethics, Polidemic, Republic of Korea, Public communication

## Abstract

This article examines the development of the Republic of Korea’s strategy to prevent the spread of COVID-19 with particular focus on ethical issues and the problem of politicization of public communication. Using prominent examples of stakeholders who have acted and expressed themselves in highly contradictory ways on the topic of the pandemic, we provide an analysis of how the public health policy discourse has entered into the realm of politicization and elaborate on the danger that this phenomenon poses in terms of rational debate and appropriate policy measures geared toward the public’s safety. Considering the role that the Republic of Korea have had in global media coverage of quarantine policies and epidemic prevention, we believe that our study makes a significant contribution to the literature because it provides a new perspective and insights into the forces at work within and around a prevention strategy that has both been lauded and seen as highly controversial.

## Introduction

In December 2019, the first report was delivered to the World Health Organization (WHO) about cases of pneumonia with unknown etiology identified in Wuhan City, China. Less than 3 months after the report, many countries were forced to implement either a full or partial lockdown. Several countries have fought to develop a vaccine and therapeutic agent for the new type of viral pneumonia, COVID-19. However, it will take a considerable amount of time for Korean people to be vaccinated and develop herd immunity. Therefore, the Republic of Korea (ROK) continues to practice a three-level social distancing scheme amid COVID-19, adjusting its social distancing drive according to the severity of the outbreak.

As a control center for disease prevention, the Korean Center for Disease Control (KCDC) recommends that citizens perform thorough personal hygiene procedures, including wearing masks and washing hands, and refrain from participating in nonessential social activities. The pandemic has led to different strategies depending on not only the political and cultural conditions of the impacted countries but also the speed, scope, or severity of the COVID-19 outbreak. The ROK has employed the “3T” strategy, namely, “Test (diagnosis/confirmation)—Trace (epidemiological survey/trace)—Treat (isolation/treatment),” successfully controlling the nationwide spread of the virus (Park et al. 2020). Such a Korean quarantine model, “K-quarantine,” including “drive-thru screening clinics” and “community treatment centers,” has been recognized as the global standard (Lee and Lee 2020). The KCDC’s strategy is to suppress the transmission of the virus through rapid and aggressive medical intervention, without some of the strict lockdown measures taken by many countries most affected by the disease. However, the ROK is also struggling to respond appropriately to the outbreak of COVID-19 and is facing ethical challenges as the duration of COVID-19 is prolonged, resulting in more casualties and economic losses, and psychological stress increases accordingly (Korea Society for Traumatic Stress Studies 2020).

This article examines how the ROK has developed the abovementioned strategy and the major ethical issues facing it in the past and present, especially regarding the issues of risk communication and the politicization of quarantine measures. First, the ROK could establish such an agile and effective quarantine system as “K-quarantine” because it made good use of the painful lessons from the MERS outbreak in 2015. One of the most serious social problems raised in connection with the MERS outbreak was the problem of trust in communication with the public. At that time, the health authorities failed to release transparent information about the virus quickly and accurately. Furthermore, in the case of COVID-19, one of the most controversial social issues in the ROK, as in other countries, is that the quarantine policy has become a target of fierce anti-governmental political struggles, not a health issue that should be approached from a medical standpoint. Just as a portmanteau of information and epidemic is “infodemic,” we propose to call the phenomenon that combines politics and epidemics “polidemic.” In the ROK, public perceptions and political reactions to COVID-19, like in many other countries, have been divided along political and ideological lines. In particular, this partisan distinction has deepened because of the close relationship between some conservative Protestant and political groups.

With no cure for COVID-19 available, the role of communication and politics is more important than ever, except to stop the spread of infection by relying on personal hygienic approaches. A crucial factor in curbing the spread of a highly contagious disease is none other than the efforts of individuals and communities to actively take infection prevention and control measures. To inspire active public participation in the pandemic preparedness process, health authorities’ messages should be objective, scientific, and reliable. Further, they need to ensure that the deepening partisan conflict between conservatives and progressives does not have a profound effect on the quarantine policies against COVID-19. The reason is, needless to say, that a virus infects anyone indiscriminately and spreads widely without political bias. Nevertheless, in our view, one of the most regrettable phenomena during the public health crisis caused by COVID-19 in Korea is the aforementioned polidemic. One of the two prominent polidemic phenomena is attributed to the physician group and the other to the religious group. We will introduce the controversial social issues related to the two groups and will subsequently discuss the core properties of a polidemic.

With regard to the first, the central question is, what are the social expectations from a group of expert physicians during a public health crisis such as COVID-19? We expect that the group will actively present ways to cope with the crisis from a scientific and medical perspective. However, the Korean Medical Association, an expert group consisting entirely of doctors, approached the pandemic politically, not scientifically. For example, as soon as the government’s medical reform plan was announced to prepare for future public health crises, the medical community strongly opposed it. The plan included increasing the number of a medical school for public health doctors. There is a widespread perception among the doctors that such new medical schools are their economic rivals and that they will serve as a factor for decrease in their income. In this crisis, they tried to achieve their goal of collective selfishness through an all-out strike against the quarantine policies that did not even make an exception for emergency room medical care. In the public health crisis related to COVID-19 in Korea, the second important polidemic phenomenon is the collusion between politics and religion. According to Article 20 of the Korean Constitution, “State religion is not recognized, and religion and State are separated.” This separation clause has been generally interpreted as not only that the State should treat all religious institutions and non-believers fairly but also that religion should refrain from intervening in politics. Until recently, politics and religion had coexisted harmoniously in the ROK, while respecting each other based on the constitutional separation principle. However, the Christian Council of Korea (CCK), an organization representing Christianity, and its former President (Park 2020), one of key figures to establish a Christian party to engage in politics using religious influence, held a large-scale rally for the anti-government struggle in collaboration with the conservative opposition. They argued that the current administration was falsely accusing Christian churches to be a source of the disease. As a leading daily newspaper shows (Korea Herald 2020), the ROK’s second wave of outbreak occurred in connection with the CCK-led anti-government rally in late August. The first wave was also caused by a particular church that did not adhere to quarantine rules. Owing to the rapid increase in the number of confirmed cases, the government increased the level of quarantine that caused enormous economic damage. We agree with many people that the freedom of association of religious assemblies should be strongly protected as a basic right of the people. However, religious leaders who dictate non-cooperation with government quarantine measures cannot protect their followers from infection.

We will differentiate between the two political actions below, which, for convenience, are called “politicization” and “politicalization.” “Politicization” refers to trying to maintain logical consistency and conducting a public justification when making political arguments. However, “politicalization” not only lacks such coherence but also does not provide public justification. The polidemic being referred to is politicization as a political phenomenon that lacks logical consistency and public justification, which is combined with the sociological characteristics of infectious diseases. We will explain in detail in “Nomenclature and Politicalization” below how the two groups mentioned above—the physician and religious groups—are participants in the polidemic.

Therefore, criticism of public health policies dealing with infectious diseases should not be subject to political controversy to the extent that it loses its logical consistency and public justification. In addition, public health authorities ought to make sincere efforts to engage public reason and deliberation suitable for a liberal democratic society. The social characteristic of COVID-19, which develops into a pandemic based on the social activities of human hosts, causes many ethical and political problems beyond mere medical ones. In such a public health crisis situation, ethics, including bioethics and public health ethics, proactively respond to such problems and boldly speculate on reasonable alternatives. Ethics is not Minerva’s owl.

After examining the past and present issues related to communicable diseases in the ROK, this paper will attempt to foreground a practical plan for how to proactively and strategically respond to COVID-19 and a future disease X.

## Transparency during Public Health Emergencies: Expensive Lessons from MERS Outbreak

With the rapid spread of a viral disease and limited knowledge of its characteristics, public health authorities, with the public’s cooperation, have no choice but to rely on traditional public health measures such as physical distancing. The value of transparency to gain the public’s trust was learned through the MERS-COV outbreak in 2015. Thus, the current framework of disease information disclosure in Infectious Disease Control and Prevention Act was shaped after this experience.

### The Outbreak of the MERS Epidemic in Korea

The ROK’s response system to COVID-19—“K-quarantine”—which successfully flattened the epidemic curve without a complete lockdown, has gained significant attention worldwide. However, for such an achievement, the ROK had to pay dearly. The country’s traumatic experience with the 2015 MERS outbreak paved the way for many successful strategies currently being developed by the government.

Starting with the first infected person in May 2015, MERS spread in the ROK as well, but the failure to respond successfully in the early stages led to the stigma of being the country with the second-highest number of MERS patients after Saudi Arabia. One of the major causes of tremendous social unrest and trillions of dollars of economic losses (Cho and Yoo 2015) is that health authorities did not disclose relevant information quickly, accurately, and transparently. The government failed to communicate with the public, causing the spread of ghost stories on the Internet and social networks (Fung et al. 2015). Unfortunately, in the early days, the government decided not to disclose the name of the MERS-related hospital because it considered unnecessary public fears and the disadvantages the hospital would suffer. However, in early June 2015, public anxiety over which hospital an MERS patient visited or was being treated in was maximized.

This is well reflected in a survey, with approximately 7 out of 10 responding that they did not trust the government’s response to MERS (Korea Herald 2015). In addition, the “One Voice Principle (MOHW 2016)” between the central and local governments was violated due to the failure of cooperation between them (Ministry of Health and Welfare 2016). The government was forced to disclose the full name of the hospital where the MERS outbreak began 18 days after the confirmation of the first infection case.

To avoid repeating the utter failure of the initial response to the MERS outbreak, the ROK government reorganized the Infectious Disease Control and Prevention Act and enacted a strengthened law as soon as the first wave of the domestic outbreak of COVID-19 occurred. According to the Act, those who refuse treatment and examination “shall be punished by imprisonment with labor for not more than one year or by a fine not exceeding 10 million won [about $90,000]” (Article 79–3), and those who refuse to be quarantined “shall be punished by a fine not exceeding three million won” (Article 80).

### Two Indicators of Trust and the Demand for Transparency According to Them

The communication guidelines of WHO (2005) translate various outbreak experiences into best practices for communication with the public—“trust,” “announcing early,” “transparency,” “understanding (the public),” and “planning” are the five practical principles, with “trust” being the most important. It is a social virtue that allows individuals to rely on others and carries the risk of being betrayed; therefore, a person who wants to be trusted cannot but demonstrate trustworthiness to others. The remaining four principles can be seen as components of trustworthiness.

The fact that trust is a matter of demonstration makes it an interactive or mutually strategic value. To say that “A” trusts “B,” for example, means that “A” makes a strategic decision under an uncertain situation. This can be explained as follows. Suppose that we are beings that perceive events in the world in a causally deterministic way like a god. Subsequently, we would not have to trust or bet on anything. Further, causally deterministic or inevitable laws need not be trusted, for doing so is useless. For example, when doing arithmetic, it is not possible to doubt or trust whether the process of doing arithmetic correctly obeys the law, given the law of arithmetic per se is undoubtedly accepted, and trust in it is not an issue. Therefore, trust is a strategic attitude one adopts because of the limitations of perceptions and understanding or the indeterminateness of the world itself. For example, if “A” trusts “B,” who is the object of trust, “A” selects the trust base that “B” has and stakes their own fate, taking on the risk of being betrayed. In other words, trusting is a strategic choice under uncertainty.

When “A” asks “B” for transparency, the reason is to know if “B” is reliable. Transparency is the instrumental value needed to determine which person or object is trustworthy.

We insist that there are two kinds of trust relationships: trust with what is and trust with who is. First, one can put confidence in an object’s “competence,” that is, how well it can do something. It is trust in what the object is or “whatness.” Second, one can put their confidence in the subject’s “ethics”—whether they can “ethically do” the job. This means that they do it in a way that cherishes and commits to a certain principle, not in an unprincipled, arbitrary, or whimsical manner. The principle here may either be utilitarian, Kantian, or a virtue ethical one. The important point is that regardless of what the principle may be, it must be one that both the trustee and the trusted person can sympathize with or willingly decide. One can trust the wholeness of the trusted object only based on this kind of principle.

Transparency can hence be seen as a value that prevents the depletion of trust and even promotes it (although the definition of trust is a controversial topic^1^ (Dawson 2009)). In the end, to gain an insightful understanding of transparency, it is essential to investigate the nature of the value of trust. According to Edelman’s (2020) Trust Barometer, the indicators on which trust is based are competence and ethics.

The demand for transparency can be divided into two categories according to these two types of indicators: one is transparency of information about how well the subject of trust has the capacity to resolve issues, and the other is transparency regarding information about whether the subject is making ethically correct decisions. The first transparency requires information about competence to trust what is and is referred to as “transparency of ‘whatness’.” The second transparency requires information about ethics to trust who is and can be called “transparency of ‘whoness’.” Transparency of whatness assumes that, in principle, the competence of the object can be measured based on “any objective indicator.” Once the level of competence is confirmed based on this objective indicator, the first transparency is secured. However, the second kind of transparency is relatively more difficult to achieve. This is because there is no “rule” to determine in advance how much they will value shared principles under uncertainty. Furthermore, it is hence not possible to measure how much the trustee will commit to the principle when functioning autonomously.

The trust may be that on governments, media companies, NGOs, corporations, health authorities, and medical professional organizations. In a public health crisis situation, trust in health authorities is extremely important. In the last MERS outbreak in 2015, Korean public health authorities failed to build trust with the public because they were not transparent in both their capacity and ethics to resolve the crisis situation.

The lack of transparency in a public health crisis can be judged as a violation of “public justification” and “public accountability” that are widely recognized as a justification requirement for public health policy (Childress et al. 2002). For example, in deciding a policy, public justification relates to providing objective grounds or reasons and public accountability to participatory joint decisions.

In their work, Childress et al. (2002) stressed the need for public health agents to “offer public justification for policies in terms that fit the overall social contract in a liberal, pluralistic democracy.” For instance, the person in charge of health authorities in coping with the MERS outbreak should have publicly justified health policy in a manner consistent with the social contract theory inherent in a liberal and pluralistic democracy, with which the ROK’s government failed to comply. Public health policy should be conducted in a manner that can be reasonably agreed upon by individuals who are free, equal, and rationally pursuing self-interest. It is public justification to provide objective grounds for reasonable agreement; thus, “transparency of whatness” is important in this situation.

On the other hand, “public accountability” means that “the public, along with scientific experts, plays an important role in the analysis of public health issues, as well as in the development and assessment of appropriate strategies for addressing them” (Childress et al. 2002). Public health officials should actively accept public accountability; they should not only sincerely seek public opinion but also seriously suggest ways to enable the public to actively participate in the formulation of public health policies.

Joint decision making is an essential strategy under uncertainty. Both parties need to know each other. This goal cannot be achieved simply by presenting an objective basis that can be confirmed. Rather, it is a matter of forming a consensus with each other and making a shared decision. “Transparency of whoness” is essential in this situation.

### The Concept vs the Conceptions of Transparency

Transparency as a procedural value, as mentioned by Angus Dawson (2009) (see footnote 1), is criticized as an opaque concept. In response to this, we propose a structure of the concept of transparency that consists of “transparency of whatness” and “transparency of whoness.” Therefore, what is the relationship between the two? It is an interactive and strategic matter to disclose to the other person how much one can be trusted. From the perspective of justice, both should be disclosed through an equally participatory method. This can be explained based on the court principle, the “Exclusionary rule,” according to which “unlawfully obtained evidence is not recognized as evidence.” This principle showcases that evidence is not simply objectively defined, as ability to testify can be recognized if the owner of the evidence can be guaranteed a participatory opportunity. In this respect, the “transparency of whoness” based on mutual consent is superior to the “transparency of whatness” as a regulatory principle.

Mark Wilson (2014) criticizes the attitude that regards transparency as a principle that can handle conflicts, such as the conflict of interest among health care professionals, as a “strong tendency to elevate transparency to the governance status of an unassailable self-evident philosophical principle.” The following example brings out the distinction between “concept” and “conception” to examine transparency more systematically. Suppose that “A” and “B” are responding to the same data with the purpose of a productive discussion. If they have a different understanding of the meaning of transparency, they have to present a concept that they can sympathize with to reach a common understanding. It is a conception that can present different interpretations of the various elements constituting the shared concept.

As we have seen so far, one of the most important features of transparency is that it is a relational and strategic value. In particular, it is a value that works in conflict of interest or even hostile relationships. Nevertheless, it is a concept that seeks common interests. Therefore, we will present a philosophically convincing concept of transparency that means “removing barriers that interfere with sharing common values or interests based on mutual understanding.” There can be various interpretations of the components that make up the concept of transparency, for example, defining the common values or interests and the kind of information to be shared. Thus, different conceptions of transparency can be established.

In the early days of the last MERS-COV outbreak, the Korean public health authorities had to pay a high price due to non-transparent policies. However, based on that lesson, it was possible to establish the relatively successful quarantine system as it is currently in place. Therefore, we propose that a deeper and more robust understanding of the role of transparency in dealing with public health emergencies, as outlined in this paper, can contribute to preparedness for any pandemic that might arise in the future.

## COVID-19 Pandemic and Polidemic

A striking problem in the ROK during the COVID-19 pandemic is that the response of health authorities has become the subject of political conflict, making rational and effective quarantine extremely difficult. In particular, the complex interaction between religious and political spheres prevents public health authorities from achieving the goal of providing safety and protecting the lives of the people. In this section, we focus on two cases: blocking entry into China and the Sarang-Jeil-Gyohoe (Sarang First Church) situation.

We will examine how crisis communication became politically contested through the response in the early stages of COVID-19. Subsequently, we will discuss the case of the Shincheonji (religious sect) infection that led to the declaration of a critical public health stage due to the explosive infection of the members, although the quarantine was sufficiently successful to cause fewer than 30 outbreaks by mid-February (Choi 2020). Finally, we would like to discuss the Sarang-Jeil-Gyohoe situation, which refers to the outbreak of a group infection at the Sarang Church, due to which social distancing, which had been mitigated to the first stage, was upgraded back to the current stage.

### Politicalization vs Politicization

First, we try to differentiate between *politicization* and *politicalization*. Politicization means that political groups oriented toward conservative or progressive political values ​​engage in discussions or struggles regarding political actions on a matter (Zürn 2014). Importantly, the participants undergo a process of public justification while keeping their political beliefs consistent. Public justification refers to trying to present a reasonable basis for one’s politics and transparently revealing it through public discourse. In general, in a democracy, society can develop further through checks, surveillance, and criticism among political groups aiming for rival or conflicting political values. Regarding the current problem, the ruling and opposition parties can criticize each other for the measures and methods adopted to cope with the new infectious disease—COVID-19—precisely because their political positions, motives, and objectives are transparent and known to the other side. Sometimes, the difference in opinion between the two positions is considerably significant that a compromise is impossible.

However, politicization is different. In our opinion, it is a political action for the absolute goal of achieving political power, although a specific political group makes claims that contradict its values ​​or the logical consistency between the policies or arguments it pursues. It simply means being instrumental. This implies that rational criticism or debate does not work according to the side-by-side “factual logic.” Furthermore, it means that the process of public justification is ignored or easily omitted. In a crisis situation such as the COVID-19 pandemic, it is extremely dangerous that, for example, the question of which quarantine model is best is not retained as an issue of politicization but instead becomes that of politicalization. It is considerably meaningful to politicalize the quarantine model we should pursue in ways such as debating whether it is herd immunity, full border blockade, or thorough medical intervention under openness. However, it is a completely different matter to try to incapacitate the public health authority without suggesting any alternatives, asserting that quarantine by the government or health authorities is a means to suppress their political claims. In other words, a political dispute is contrary to public accountability in that it excludes or ignores the opinions of the “opposite group” rather than admitting them as the differing opinions, for the very reason that they are the opinions of the opposing group and conflict with one’s own.

### Nomenclature and Politicization

COVID-19, before the official establishment of a global term, was known as “Wuhan pneumonia” at the outset. However, the WHO officially named this epidemic “COVID-19.” The reason is to prevent persistent prejudice and discrimination against the area and the people living there. However, the problem in the ROK was that despite this name change, some political groups and (especially) conservative media insisted that “Wuhan pneumonia” should be used and claimed that the government, which did not do so, was driving “China toadyism (Sadaejuui).”^2^ The point driven was that quarantine policy was an example of the government’s attitude of adhering to the frame of China’s superior powers and that it had nothing to do with the medical purpose of quarantine. This resulted in undermining the health authorities, thereby lowering the trust in health policy. In addition, some conservative medical groups have urged the government to block the entry of Chinese citizens. In short, whether to use epicenter names when naming infectious diseases in such a public health crisis is an important issue worthy of debate. Therefore, *politicalization* can be productive and desirable as it allows a political group to present a basis for and reasonably defend its argument to justify it publicly. Of course, specifying the epicenter of the epidemic in the early stages of an infectious disease can help to some extent in devising a quarantine plan. However, interestingly, the same group of doctors demanding blocking entry of people from China has made no such demand regarding the USA, where the number of confirmed cases is exploding. In addition, it was difficult to find an article in which the same media that strongly pressed for blocking the entry of China criticized the Korean government for not doing the same for Americans. If it was not a “decision for political conflict” but a judgment based on medical and scientific grounds that the entry to China should be blocked, it should have insisted that countries other than China, where the confirmed cases are prevalent, should be blocked simultaneously. If a critic disparaged the government for “Chinese toadyism,” that person should have criticized it for “American toadyism” as well. Losing the logical coherence of a policy and neglecting its justification is difficult to regard as a politicization and an attitude of valuing public legitimacy.

The logical inconsistency of the arguments made by some political groups and the media themselves occurred even in the situation of the exponential increase of COVID-19 cases in Daegu. As the religious group Shincheonji did not observe quarantine rules, the infection started in Daegu spread nationwide, resulting in the critical situation in which the health crisis was elevated to a serious stage. However, some conservative political groups and media criticized the connection drawn between Daegu and Corona and condemned the health authorities and the government within the frame of “regional discrimination” regarding government documents in which the name “Daegu Corona” was inadvertently used. However, if their argument that revealing the epicenter of the epidemic is more advantageous in preventing the spread of infection is correct, then according to their logic, the term “Daegu Corona” should have been used, just like “Wuhan pneumonia.” However, unlike in the case of the latter term, this time, highlighting the region was actively criticized. Furthermore, it was difficult to find evidence that the political group or the media actively promoted the work to present a reasonable basis for such a change in attitude. In other words, it can be seen as neglecting public justification. The reason for this can be guessed easily as it is based on the logic of regional sentiment, which has been one of the key factors governing Korean politics to date. It is because the area in question is one where conservative forces continue to dominate, accounting for a large proportion of the population.

### Sarang-Jeil-Gyohoe Case

The public health situation in Korea is facing a second peak in newly confirmed cases due to a group infection centered on the Sarang-Jeil Gyohoe (Church). In a few days, 700 confirmed cases were identified, and the government raised social distancing from stage 1 to stage 2 and is preparing to issue stage 3. The outbreak of this large-scale infection can be traced to an anti-government rally held at Gwanghwamun Square on Liberation Day (15 August). Pastor J, who was in charge of the church and is known to have led anti-government protests, led this rally too. Over this rally, the passport insists on the protection of or support for the opposition party, and against such a passport, the opposition party is refusing to be weak and unjustifiable argument.

Health policy in a pandemic is a political responsibility, and therefore, must be politicized as the object of reasonable criticism. Nevertheless, the establishment and implementation of health policies must be *de-*politicized. A concern in the recent development of the health crisis in Korea is that health policies are becoming politically contested, for example, through conspiracy theories on the determination of confirmed cases, which undermines the authority of health officials and ultimately lowers trust in health policy. Thus, it undermines the citizens’ voluntary compliance with quarantine and hinders active participation of the community, thereby creating a situation in which the whole country has to bear the damage. The fact is that when some deviant or immoral behavior occurs as a result of a particular evaluation of the nature of the infectious disease that is based on political faction logic, a health crisis situation, wherein the coronavirus spreads widely, can arise due to the inability to properly control such behavior.

In the absence of medical means to cope with new infectious diseases, it is necessary to reestablish “medical” means, rather than relying on simple information transmission. Political controversy prevents communication from becoming “medical” and degrades trust in scientific messages. It prevents these messages from dealing with thorough medical and scientific treatment. As seen in this period, a problem faced during the outbreak was the infodemic caused by misinformation, fueled by anxiety and fear. However, the political disagreement related to the disease, which makes communication excessively controversial, is equally worrisome. The politicization of a disease is a considerably serious problem as it leads to the loss of health officials’ authority according to political gains and losses, thereby fundamentally undermining the trust in quarantine policies.

#### Conclusion

In the absence of medical means to cope with new infectious diseases, communication between experts such as health authorities, government officials, political and medical groups, and the public should be uncontroversial, “medical” communication. Furthermore, this medical communication should be capable of maintaining or promoting solidarity in a way that is not directly or indirectly related to the exclusion and stigma of certain groups, including that caused by regional discrimination. Pandemics such as COVID-19 create a social environment where personal privacy is violated, freedom of movement is restricted, and business activities for making a living are restricted. In particular, the possibility of contagion leads to a situation that threatens one’s own life and safety. In such a pandemic situation, the dark aspects of human psychology are more clearly revealed, which can make it difficult for us to make rational and moral judgments due to partisan strategies.

At times like this, we should pay more attention to whether the communication and social messages of the leading group are politically biased. If a socially responsible organization or group ignites a partisan-fueled message, disguised as a social message in the name of preventing infectious diseases, that message is not only counter-productive in preventing infectious diseases but can also threaten our lives and safety.

## Footnotes

1 Angus Dawson raises concerns that “the appeal to transparency is unclear” and says, “despite its ubiquity in contemporary bioethics, I’m not sure what the term transparency means. After all, transparency is just a metaphor suggesting that we make things visible. It is not, in itself, either a moral principle or a key part of any substantive moral theory.” (Dawson 2009).

2 Sadaejuui is Korean term for a cultural attitude, wherein the weaker nation tries to adopt the politically strong State’s perspective as its own. It can be translated as “serving-the-great-ism” (Wikipedia).
